# Data-independent acquisition-based mass spectrometry(DIA-MS) for quantitative analysis of patients with chronic hepatitis B

**DOI:** 10.1186/s12953-023-00209-6

**Published:** 2023-06-06

**Authors:** Bo Wang, Qian Zhang, Lili Wu, Cunliang Deng, Meiyan Luo, Yu Xie, Gang Wu, Wen Chen, Yunjian Sheng, Peng Zhu, Gang Qin

**Affiliations:** 1 Department of Infectious Diseases, The Affiliated Hospital of Southwest Medical University, Luzhou, 646000 Sichuan China; 2 Department of Gastroenterology, Suining First Pepole’s Hospital, Suining, 629000 Sichuan China; 3 College of Graduate, Southwest Medical University, Luzhou, 646000 Sichuan China

**Keywords:** Chronic hepatitis B, Hepatitis B virus, CHB, DIA-MS

## Abstract

Chronic hepatitis B is a significant public health problem and complex pathologic process, and unraveling the underlying mechanisms and pathophysiology is of great significance. Data independent acquisition mass spectrometry (DIA-MS) is a label-free quantitative proteomics method that has been successfully applied to the study of a wide range of diseases. The aim of this study was to apply DIA-MS for proteomic analysis of patients with chronic hepatitis B. We performed comprehensive proteomics analysis of protein expression in serum samples from HBV patients and healthy controls by using DIA-MS. Gene Ontology (GO) terms, Kyoto Encyclopedia of Genes and Genomes (KEGG) pathways, and protein network analysis were performed on differentially expressed proteins and were further combined with literature analysis. We successfully identified a total of 3786 serum proteins with a high quantitative performance from serum samples in this study. We identified 310 differentially expressed proteins (DEPs) (fold change > 1.5 and *P* value < 0.05 as the criteria for a significant difference) between HBV and healthy samples. A total of 242 upregulated proteins and 68 downregulated proteins were among the DEPs. Some protein expression levels were significantly elevated or decreased in patients with chronic hepatitis B, indicating a relation to chronic liver disease, which should be further investigated.

## Background

According to the latest WHO estimates, more than 257 million people experience chronic hepatitis B virus (HBV) infection worldwide [1]. HBV infection results in approximately 887,000 deaths every year [1]. Chronic hepatitis B(CHB) is usually characterized by the presence of detectable hepatitis B surface antigen (HBsAg) lasting for longer than 6 months, including HBeAg-positive CHB and HBeAg-negative CHB [2]. Only 10.5% of people with CHB infection are diagnosed, and 17% of them are on treatment [3]. The annual incidence of cirrhosis inpatients with CHB without antiviral therapy is 2–10%, and hepatocellular carcinoma in patients with cirrhosis is 3–10% [4–8]. HBV infection can cause both acute and chronic disease, including fulminant liver failure, acute-on-chronic liver failure, cirrhosis, and even hepatocellular carcinoma. Therefore, CHB is the leading cause of cirrhosis and the most important cause of hepatocellular carcinoma worldwide [9].

The pathological mechanism of CHB is very complex, and when HBV is chronically infected, many aspects of CD8 + T cells are depleted [10, 11], such as their decreased cell proliferation capacity, and significantly reduced ability to secrete IFN-γ, IL-2, TNFα, granzyme, and perforin. Soluble programmed death ligand 1 (sPD-L1) regulates T-cell depletion [12], and PD-1/PD-L levels [13] are associated with inflammatory responses to CHB, indicating that immunomodulation plays an important role in the pathogenesis of CHB. Similarly, studies have shown that IL-1β [14] is associated with the degree of inflammation in the liver in patients with CHB. However, the pathogenesis of CHB has not been fully elucidated.

Blood is considered a very important source of disease-related biomarkers [15], and the supernatant remaining after isolating blood cells and fibrin is an ideal sample for bioanalysis, as it retains a wealth of biological information, and serum proteomics is essential for disease biomarker discovery research [16, 17]. Circulating proteomic panels can be used for diagnosis and risk stratification of acute-on-chronic liver failure in patients with viral hepatitis B [18]. Data-independent acquisition mass spectrometry (DIA-MS) is a label-free quantitative proteomics method that enables deep proteome coverage and precise relative quantification in a single liquid chromatography [19]. DIA-MS analysis has been successfully applied to the study of a wide range of diseases, including pancreatic cancer [20], Parkinson’s disease [21] and aortic stenosis [22]. In this study, serum proteins from CHB patients and healthy controls were quantitatively analyzed by DIA-MS technology, and Gene Ontology (GO) terms, Kyoto Encyclopedia of Genes and Genomes (KEGG) pathways, and protein network analysis were performed on differentially expressed proteins, and further combined with literature analysis.

## Materials and methods

### Clinical sample collection

According to the diagnostic criteria of CHB [23] and the exclusion criteria (other types of viral hepatitis, autoimmune liver diseases, alcoholic liver disease, drug-induced hepatitis, malignant tumors, other organ failure and psychiatric diseases), ten clinical samples were collected from the Department of Infectious Disease at the Affiliated Hospital of Southwest Medical University (Luzhou, China) in this study. Briefly, 5 patients with chronic hepatitis B (CHB, *n* = 5) and 5 healthy volunteers ( normal controls, NC, *n* = 5) were enrolled, and clinical data, including the patient’s medical history, physical examination and biochemical examination, were obtained. Serum samples of peripheral blood (5 ml) were collected from patients with CHB before they received treatment and from healthy controls, centrifuged at 4000 × *g* for 10 min and stored at − 80 °C until further proteomic analysis. The Ethics Committee of the Affiliated Hospital of Southwest Medical University approved the study (KY2021014). This trial was registered at the Chinese Clinical Trial Registry (www.chictr.org.cn) (trial registration number ChiCTR2100042896). All patients were over 18 years old and signed the corresponding informed consent form. This study was carried out in accordance with all the guidelines and principles stated in the Declaration of Helsinki.

### Protein extraction, protein enrichment, and protein enrichment quality control

SDS-free lysate was added to 100 μL of serum samples and finally made up to a total volume of 1 ml.Then, the proteins were reduced and alkylated to disrupt the disulfide bonds as follows: a) DTT was added to samples at a final concentration of 10 mM, and the samples were incubates at 37 °C for 30 min. b) Iodoacetamide was added to the samples at a final concentration of 55 mM and incubated in the dark at room temperature for 30 min. c) The mixture of proteins was passed through a solid phase extraction (SPE) C18 column for protein enrichment.

The the treated protein solution was enriched by C18 column SPE (activating, conditioning, loading, washing, eluting, drying). The drained proteins were redissolved in a solution of 20 μL of 50 mM ammonium bicarbonate according to the instructions of the Pierce Quantitative Fluorometric Peptide Assay. An appropriate amount of protein solution was added to each sample, which was then mixed with an appropriate amount of sample buffer, heated at 95 °C for 5 min, and centrifuged at 25,000 × g for 5 min, The supernatant was loaded into a 12% SDS polyacrylamide gel, subjected to 80 V constant pressure electrophoresis for 30 min, and then subjected to 120 V constant pressure electrophoresis for 120 min. After electrophoresis, the gel was stained and destained by a protein staining instrument for 10 min and the images were scanned.

### Peptide fractionation(high pH reversed-phase separation)

The LC-20AB HPLC system (Shimadzu, Japan) coupled to a high pH C18 column (Gemini, 5 μm, 4.6 × 250 mm) was used. An equal amount of peptides from each sample was taken to pool a mixture, and 20 μg of mixture was diluted with 2 mL of mobile phase A (5% ACN, pH 9.8). The sample was placed on the column and then eluted through a gradient at a flow rate of 1 mL/min. The protocol was as follows: 5% mobile phase B (95% ACN, pH 9.8) for 10 min, 5% to 35% mobile phase B for 40 min, 35% to 95% mobile phase B for 1 min, flow phase B for 3 min, and 5% mobile phase B for 10 min. The elution peak was monitored at a wavelength of 214 nm, and one component was collected per minute, the samples were combined according to the chromatographic elution peak map to obtain 10 fractions and were freeze-dried.

### Mass spectrometry-based proteomics analysis

The drained peptide samples were reconstituted with mobile phase A (2% ACN, 0.1% FA) and centrifuged at 20,000 × g for 10 min, and the supernatant was taken for injection. Separation was carried out by an UltiMate 3000 UHPLC liquid chromatograph (Thermo Fisher Scientific, San Jose, CA). The sample was first enriched in the trap column and desalted and then entered a tandem self-packed C18 column (150 µm inner diameter, 1.8 µm column particle size, approximately 35 cm column length) and separated at a flow rate of 500 nL/min through the following effective gradient: 0–5 min, 5% mobile phase B (98% ACN, 0.1% FA); 5–90 min, mobile phase B rose linearly from 5 to 25%; 90–105 min, mobile phase B increased from 25 to 35%; 105–110 min, mobile phase B increased from 35 to 80%; 110–115 min, 80% mobile phase B; 115–120 min, 5% mobile phase B. The nanoliter liquid phase separation end was directly connected to the mass spectrometer with the following settings.

The liquid-separated peptides were ionized by nanoESI and entered the tandem mass spectrometer Q-Exactive HFX (Thermo Fisher Scientific, San Jose, CA) for data-dependent acquisition (DDA) detection mode.. The DDA parameter settings were as follows: ion source voltage 1.9 kV; MS scan range 350–1,500 m/z; MS resolution 120,000; maximum ion implantation time (MIT) 50 ms; secondary MS/MS collision type HCD (HCD-MS/MS); collision NCE 28; MS resolution 30,000; MIT 100 ms; and the dynamic exclusion time 30 s. The starting m/z for MS/MS was fixed at 100. Precursors for MS scan satisfied the following criteria: charge 2 + to 6 + , and among the top 20 precursors an intensity over 2E4. The AGC was MS 3E6, and MS/MS 1E5.

The liquid-separated peptides were ionized by nanoESI and injected into the tandem mass spectrometer Q-Exactive HFX (Thermo Fisher Scientific, San Jose, CA) for in data-independent acquisition (DIA) detection mode. The main parameter settings were as follows: ion source voltage 1.9 ~ 2 kV; first-stage MS (mass spectrometry) scanning range 400 ~ 1250 m/z; MS resolution 120,000; maximum ion implantation time (MIT) 50 ms; the 400–1250 m/z range was equally divided into 45 continuous window fragments, and the signal was acquired. The ion fragmentation mode was HCD, the MIT was selected as automatic mode, the fragment ions were detected in Orbitrap, the resolution was 30,000, and the fragmentation energy was distributed fragmentation: 22.5, 25, and 27.5; AGC was 1E6.

### Mass spectrometry analysis

This study was executed using MaxQuant (http://www.maxquant.org) [24] for identification of DDA data and served as a spectrum library for subsequent DIA analysis. The analysis used raw data as input files, set corresponding parameters and databases, and then performed identification and quantitative analysis. The identified peptides FDR (false discovery rate) ≤ 1% were used to establish the final spectral library. The DIA data were analyzed using the iRT peptides for retention time calibration. Then, based on the target-decoy model applicable to SWATH-MS, the false positive control was completed at 1% FDR to obtain significant quantitative results. MSstats [25] is an R package that can be used for statistical evaluation of significant differences in proteins or peptides. Proteins with a fold change ≥ 1.5 and *P* < 0.05 were considered to be differentially expressed proteins (DEPs).

### Bioinformatics and statistical analyses

To further understand the functions of the DEPs in CHB, these DEPs were assessed by GO analysis (http://geneontology.org/). The signaling pathways of these DEPs were enriched and analyzed by using the KEGG pathway database (https:/kegg.jp/). All DEPs were compared with the KOG database (https://mycocosm.jgj.doe.gov/help/kogbrowswe.jsf/), and the corresponding KOG annotation results were obtained. PPI network analyses were applied to find the interactions among all DEPs by using the STRING database 11.0 (https://string-db.org/). The DEPs in the centre of the network between the normal group and the CHB group were clarified.

The patients’ clinical information and ELISA data were analyzed with SPSS 21.0 software. Measurement data are described as the mean ± standard deviation, and counting data are presented as percentages (%) using the Mann–Whitney U test or unpaired Student’s t test. Furthermore, the chi-squared or Fisher exact tests were used to compare the categorical variables. *p* values < 0.05 were considered statistically significant.

## Results

### Clinical characteristics of serum samples

A total of 10 serum samples from CHB patients and healthy controls were used for proteomic analysis, and there were differences in basic data between the CHB group and the healthy group (Table 1).

**Table 1:** Baseline characteristics of patients enrolled

Characteristic	Health control (*n* = 5)	CHB (*n* = 5)	*P*
Gender(male/female)	3/2	1/4	1.00
Age (years)	34.20 ± 4.27	42.80 ± 9.6	0.11
Laboratory parameters			
WBC (10^9^/L)	5.87 ± 1.13	4.72 ± 1.50	0.03
NEU (10^9^/L)	3.23 ± 0.63	2.98 ± 0.91	0.63
HGB (10^9^/L)	145.60 ± 14.26	147.80 ± 9.09	0.78
PLT (10^9^/L)	265.40 ± 80.77	154.40 ± 42.07	0.03
ALT (IU/L)	22.14 ± 10.63	782.54 ± 168.88	0.00
AST (IU/L)	17.92 ± 2.07	429.08 ± 131.67	0.00
ALB (g/L)	46.18 ± 2.59	44.18 ± 2.63	0.26
TB (umol/L)	10.26 ± 3.98	24.42 ± 11.88	0.04
DB (umol/L)	3.34 ± 1.14	9.70 ± 4.40	0.01
ALP (umol/L)	23.84 ± 6.14	137.86 ± 80.79	0.00
CER (umol/L)	66.44 ± 8.63	70.00 ± 14.81	0.66
INR	N/A	1.07 ± 0.07	N
HBV-DNA (Log)	0	4.03 ± 3.96	N
K	N/A	3.93 ± 0.32	N
NA	N/A	139.40 ± 1.34	N
CL	N/A	106.16 ± 2.46	N
AFP	N/A	11.07 ± 9.56	N

*P* value < 0.05 for comparisons between CHB and Health control

### Identification of DEPs

**Fig. 1:**
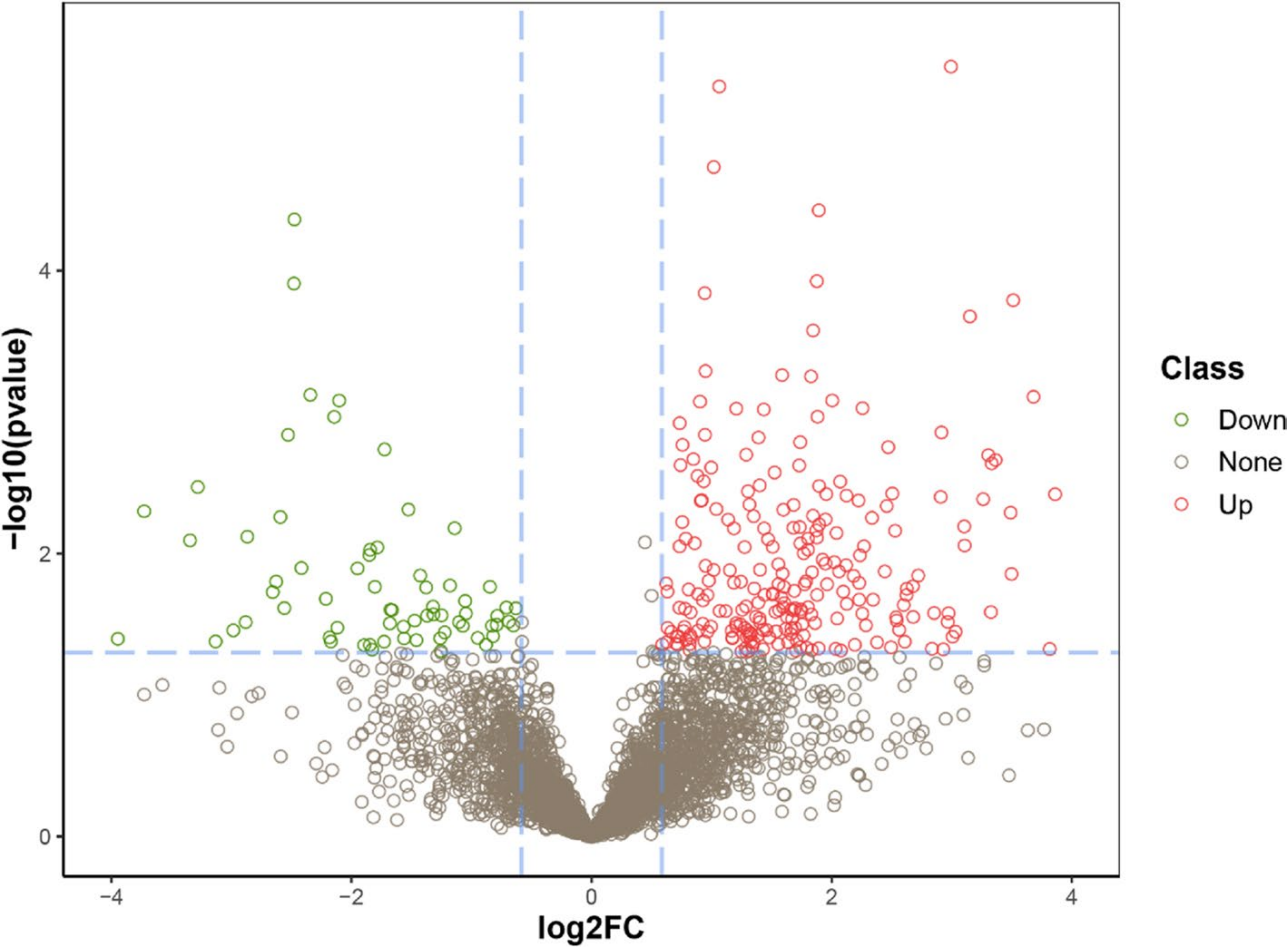
Volcano plot (where the x-axis represents the log2 of the normalized fold change, and the y-axis represents the negative decadic logarithm of the significance value) of the differentially expressed proteins between the CHB group and the health control group. Red represents significantly upregulated proteins, green represents significantly downregulated proteins, and gray represents unchanged proteins

In this study, we compared the serum proteome profiles of the CHB group and the control group. Quantification of proteins was performed based on a fold change > 1.5 and *P* value < 0.05 as the criteria for a significant difference. DEPs were assessed by using the MSstats software package. A total of 310 proteins were identified with differential expression between the CHB group and the control group, of which 242 proteins were upregulated and 68 proteins were downregulated (Fig. 1). The results visually reflected a significant difference in protein expression between the CHB group and the healthy control group. The volcano plot directly shows the expression intensity of these proteins.

### GO enrichment analysis

**Fig. 2:**
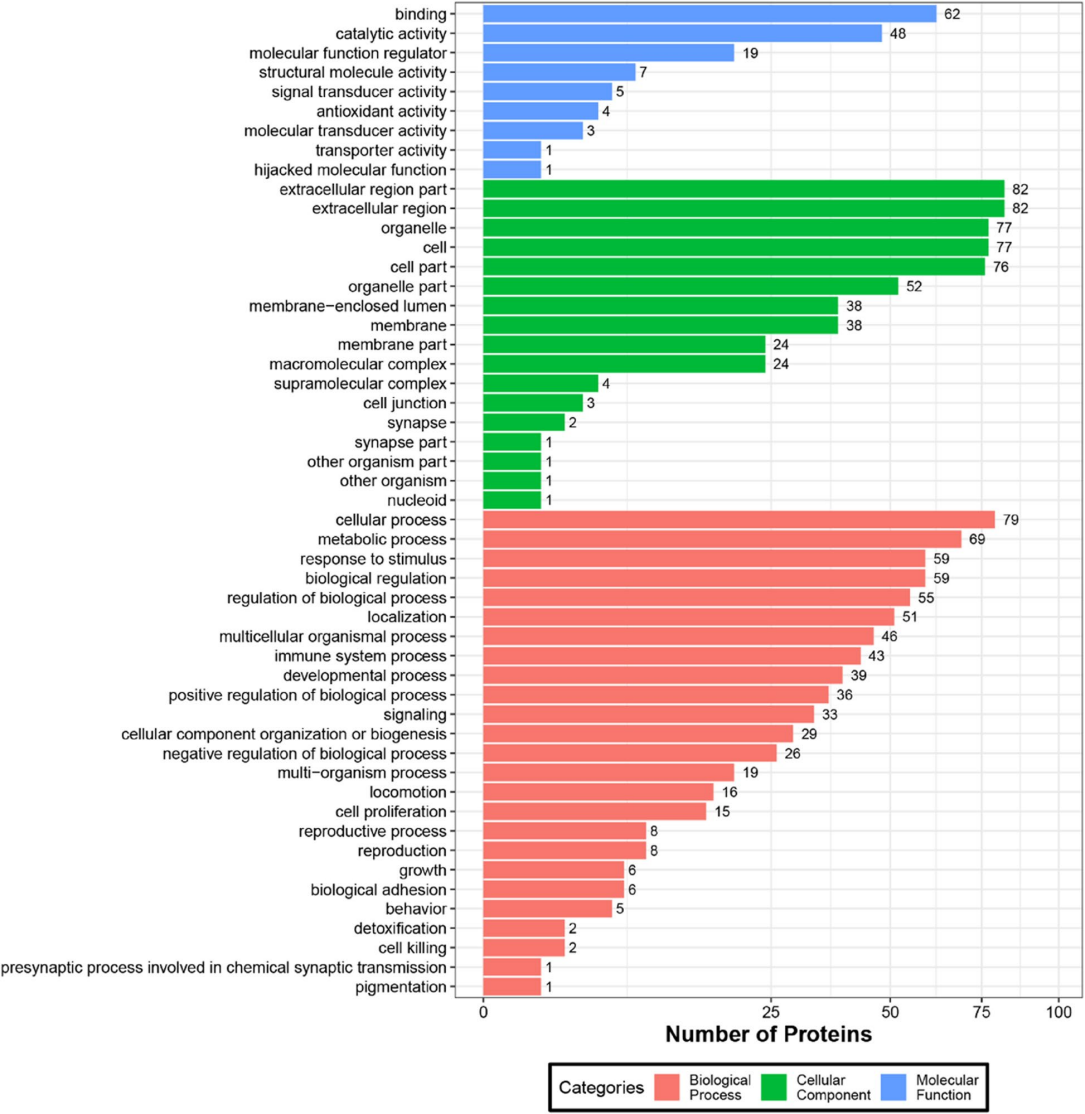
GO functional classification of DEPs. The x-axis represents the number of DEPs. The y-axis represents GO terms. All GO terms are grouped into 3 ontologies: biological process, cellular component and molecular function; blue indicates molecular function, green indicates cellular component, and orange indicates biological process

**Fig. 3:**
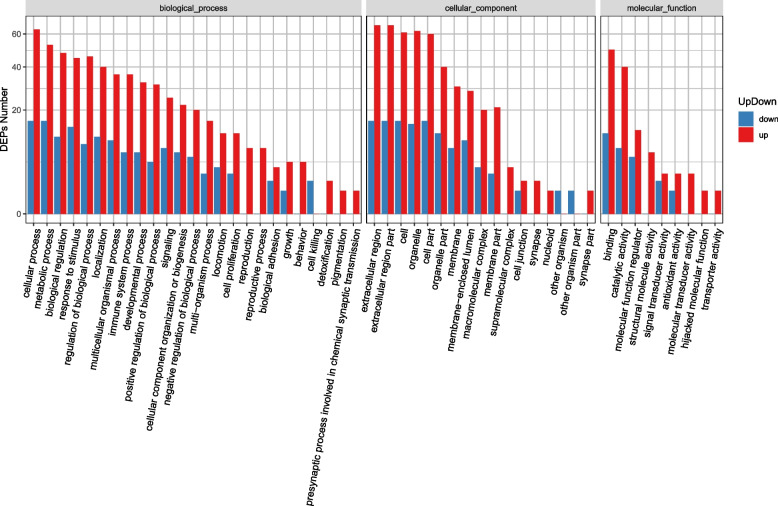
GO functional classification between upregulated proteins and downregulated proteins.The x-axis represents GO terms.The y-axis represents the number of DEPs. Red indicates upregulated proteins, and blue indicates downregulated proteins

We performed GO enrichment analysis on 310 differentially expressed proteins. The results showed that 242 upregulated proteins and 68 downregulated proteins have potential functions. GO functional classification maps of all differentially expressed proteins, with upregulated proteins and downregulated proteins distinguished (Figs. 2, 3). GO has a total of three ontologies, which describe the molecular function, cellular component, and biological process of genes. Upregulated proteins and downregulat proteins were involved in cellular process, metabolic process, response to stimulus, biological regulation, regulation of biological process, localization, multicellular organismal process, immune system process, and so on. This result indicated that the molecular mechanisms may differ between the CHB group and the healthy control group. In addition, these differentially expressed proteins were also involved in molecular function binding, catalytic activity, and molecular function regulation. The cell component is also involved, specifically the extracellular region part, extracellular region, organelle, cell, and cell part. However, some separated enrichment of upregulated proteins (reproduction, reproductive process, behavior, supramolecular complex, synapse, and molecular transducer activity) or downregulated proteins (cell killing) was observed.

### KOG classification

**Fig. 4:**
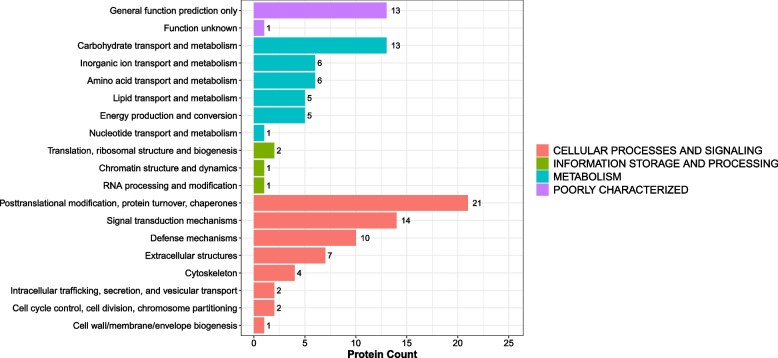
Histogram presentation of KOG classification. The x-axis represents the number of DEPs, and the y-axis represents the KOG classification entry

Eukaryotic orthologous groups (KOGs) are databases for direct homologous classification of proteins. The analysis compares the identified proteins with the KOG database (Fig. 4), predicted the possible functions of these proteins, and generated functional classification statistics. The most representative KOG category was cellular processes and signaling, with main associations of differential proteins being posttranslational modifications, protein turnover, chaperone activity, signaling, and defense mechanisms.

### KEGG enrichment analysis

**Fig. 5:**
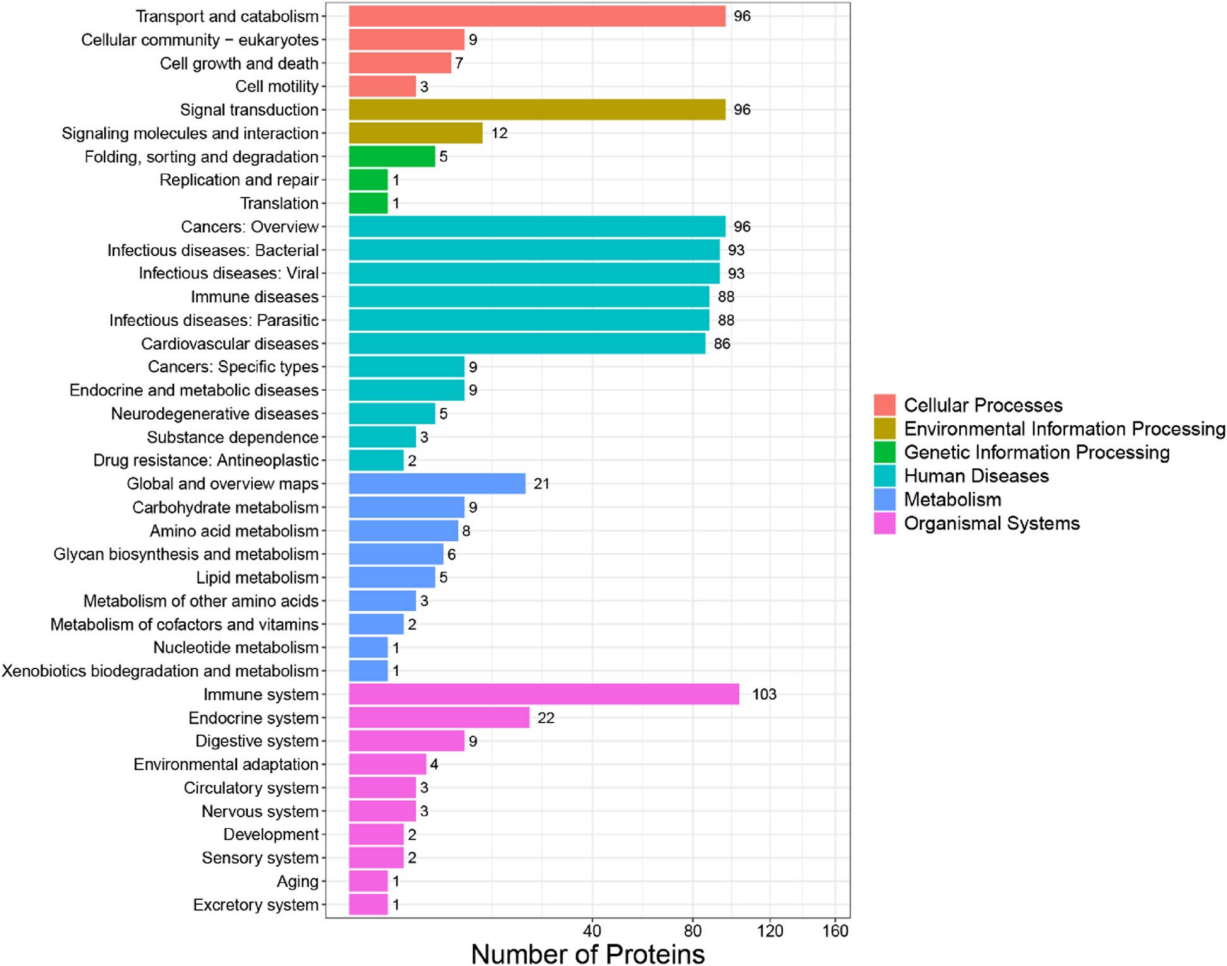
Differential protein pathway classification statistical chart. The x-axis represents the number of differentially expressed proteins, and the y-axis represents the pathway annotation entry

**Fig. 6:**
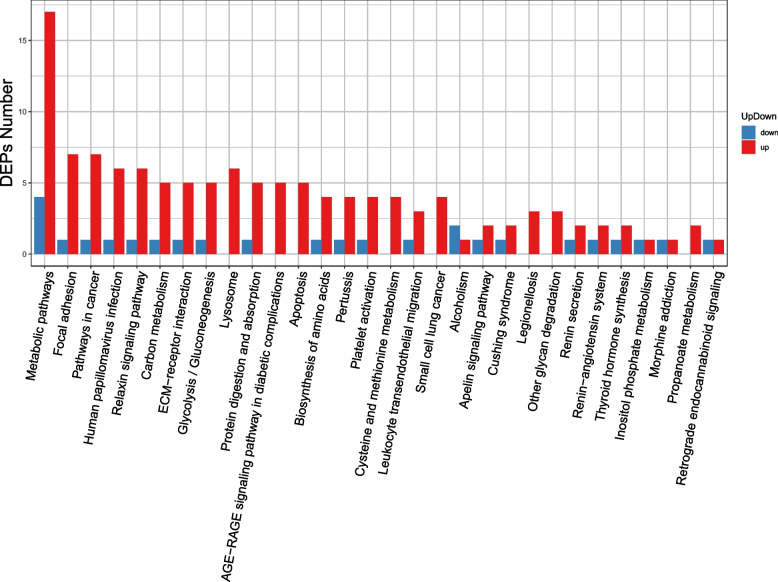
Differential protein pathway classification statistical chart. The x-axis represents the pathway annotation entry, and the y-axis represents the number of proteins. Red indicates upregulated proteins, and blue indicates downregulated proteins

Next, we performed KEGG enrichment analysis to further identify differential protein biological functions and to distinguish upregulated proteins and downregulated proteins (Figs. 5, 6). Upregulated differentially expressed proteins were annotated to a total of 30 pathways, where the main pathway was the metabolic pathway: downregulated differentially expressed proteins ware annotated to 22 pathways, and the most important pathway was also the metabolic pathway.

### Protein‒protein interaction network

**Fig. 7:**
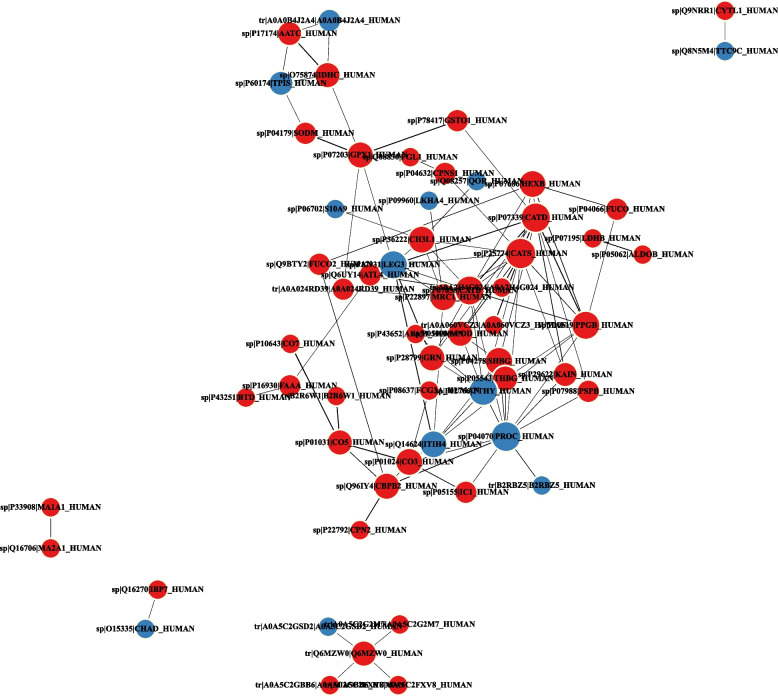
Differential protein interaction network diagram. Red indicates upregulated proteins, and blue indicates downregulated proteins. The size of the circle indicates the density of the relationship

Proteins usually interact with each other to perform their functions after binding as complexes. Next, the differentially expressed proteins were imported into the STRING database (STRING 11.0), DEPs were analyzed by comparison with the STRING [26] protein interaction database, and the network interaction diagram (Fig. 7) was drawn by taking the interaction relationship of the top 100 proteins based on the confidence level. The most strongly upregulated differential protein relationships were with CATD (cathepsin D), CATES (cathepsin S), CATB (cathepsin B), and PGRN (progranulin). The most strongly downregulated differentially expressed protein relationships were with LEG3 (galectin-3), and ITIH4 (inter-alpha-trypsin inhibitor heavy chain H4).

## Discussion

Chronic infection with HBV, characterized by high morbidity and mortality sequelae, is the leading cause of both acute and chronic liver disease, including fulminant liver failure, acute-on-chronic liver failure, cirrhosis, and even hepatocellular carcinoma. CHB is still a major global health problem [9]. The pathogenic mechanism of CHB is complex and unclear. Metabolomics and proteomics studies have revealed that some proteins can reflect deficiencies in hematological functions and are highly associated with HBV-related acute-on-chronic liver failure progression [18]. Another study revealed that some proteins were highly associated with significant fibrosis in patients with nonalcoholic fatty liver disease [27].

In this study, we successfully identified a total of 3786 serum proteins with a high quantitative performance from CHB patient serum samples using DIA-MS proteomic analysis. By bioinformatics analysis, we found 310 DEPs between chronic Hepatitis B patients and healthy controls. The GO analysis and KEGG analysis showed that 242 upregulated proteins and 68 downregulated proteins have potential functions. DEPs were enriched in immune-rated pathways and metabolic pathways. Immunity plays an important role in CHB. Liver damage during CHB occurs mostly through immunological processes [28].

Combined with the results of the protein‒protein interaction network, our results indicated that the expression of cathepsins (cathepsin B, cathepsin D, and cathepsin S) and PGRN was significantly higher than that in healthy controls. Cathepsin D, a soluble lysosomal aspartic protease has many biological functions and is involved in the degradation of proteins, regulation of cell death, and activation of inflammatory cells and plays a crucial role in promoting cancer invasion, metastasis, and angiogenesis [29, 30]. In some studies, Currently, circulating cathepsin D levels in nonalcoholic fatty liver disease were shown to be useful for the assessment of disease severity [31]. In our study, GO enrichment of differentially expressed proteins revealed that cathepsin D mainly functions in collagen catabolic process and extracellular region, and KEGG enrichment analysis of differentially expressed proteins showed that cathepsin D is mainly involved in lysosomes, while KOG classification analysis of differentially expressed proteins showed that cathepsin D is involved in posttranslational, protein turnover, and chaperones. Evidence has shown increased cathepsin D in human liver tissues with chronic HBV infection [29]. Cathepsin B and cathepsin S are cysteine proteases that play important roles in various physiological and pathological processes. Cathepsin B was increased in the fibrotic liver and was significantly correlated with hepatic hydroxyproline levels. This result suggests that cathepsin B is induced by hepatic collagen levels and is implicated in degrading collagens [32]. A previous study reported that cathepsin B plays a key role in hepatocellular apoptosis and liver injury and mediates liver cancer cell apoptosis contributing to inflammation and fibrogenesis [33]. Cathepsin S plays a key role in tumor invasion and metastasis. A recent study demonstrated that cathepsin S induced apoptosis of hepatocellular carcinoma cells and increased their chemosensitivity by regulating nuclear factor kappa-B and activating cleaved caspase-3 [34]. PGRN (progranulin), a secretory glycoprotein, has many biological functions involved in tissue development, regeneration, inflammation, metabolic disease, and neurodegeneration [35]. A study demonstrated that PGRN improved inflammation and fibrosis and reduced steatosis and hepatocellular injury in mouse models of hepatic fibrosis and nonalcoholic steatohepatitis. PGRN showed protective effects against hepatic injury, inflammation, and fibrosis by downregulating the inflammatory response [35].

In this study, our results indicated that the expression of LEG3 (galectin-3), ITIH4(inter-alpha-trypsin inhibitor heavy chain H4), was significantly lower than that in healthy controls. Galectin-3, a member of the galectin family, has many biological functions involved in cell growth, apoptosis, differentiation, inflammation, fibrosis, and the pathogenesis of autoimmune and inflammatory processes [36]. Galectin-3 expression levels were significantly increased in tumor tissue and serum, and in vitro and in vivo studies indicated that galectin-3 can facilitate hepatoma cell proliferation and reduce apoptosis among these cells [37]. A study indicated that serum galectin-3 levels in CHB patients were decreased compared with those in patients with hepatocellular carcinoma and cirrhosis and confirmed that there was no difference in serum galectin-3 levels between patients with hepatocellular carcinoma and patients with cirrhosis [38, 39]. In this study, serum galectin-3 levels in CHB patients were decreased compared with those in healthy controls. The biological function of galectin-3 in chronic viral hepatitis B has remained unknown.

ITIH4 is a plasma glycoprotein that belongs to a family of proteins called the inter-alpha-trypsin inhibitor family. Many studies have found that ITIH4 is involved in chronic liver diseases, including chronic viral hepatitis, autoimmune liver disease, and hepatocellular carcinoma [40–42]. Previous research has shown that the serum levels of ITIH4 increased successively as fibrosis progressed in children with chronic hepatitis C [40]. However, another study confirmed that the serum levels of ITIH4 decreased as fibrosis progressed in patients with chronic hepatitis C [43]. In this study, we showed that ITIH4 was highly decreased in patients with CHB.

In conclusion, we successfully identified a total of 310 proteins in serum samples of patients with CHB. Some protein expression levels were significantly elevated or decreased in patients with CHB, indicating a relation to chronic liver disease. Furthermore,, in CHB patients, some proteins should be further investigated.

## Data Availability

All the proteomic/phosphoproteomic raw data and the results files of EESCC and EDAC had been uploaded to the iProx Consortium (https://www.iprox.org/) with the PXD identifiers (PXD026736).
